# Pain and Oral Health Related Quality of LIfe among Patients Undergoing Fixed Orthodontic Treatment: A Descriptive Cross-sectional Study

**DOI:** 10.31729/jnma.4817

**Published:** 2020-06

**Authors:** Prakash Poudel, Sirjana Dahal, Vivek Bikram Thapa

**Affiliations:** 1 Department of Orthodontics and Dentofacial Orthopaedics, Kathmandu Medical College, Duwakot, Bhaktapur, Nepal; 2 Department of Community and Public Health Dentistry, Kathmandu Medical College, Duwakot, Bhaktapur, Nepal

**Keywords:** *fixed orthodontic treatment*, *oral health impact profile-14*, *pain*

## Abstract

**Introduction:**

Fixed orthodontic procedures such as separator placement, archwire placement and activations, application of orthopaedic forces, and debonding of brackets produce pain in patients. This study was conducted to assess pain and oral health-related quality of life among patients undergoing orthodontic treatment.

**Methods:**

This descriptive cross-sectional study was conducted among 152 orthodontic patients of a teritary care center from January 2019 to October 2019 after receiving ethical approval from the Institutional Review Committee (Ref. no. 2311201813). Convenience sampling method was done to select the participants. Oral health-related quality of life using "Oral Health Impact Profile-14" and pain experienced during the first month of fixed orthodontic treatment were assessed. Data analysis for calculation of frequency and proportion was done in Statistical Package of Social Sciences.

**Results:**

Mean pain score of the study participants was 5.05±2.07 and their mean oral health impact was 12.71±7.27. Most of the study participants 86 (56.58%), had experienced moderate pain due to orthodontic treatment. Out of the reported impacts, 134 (88.2%) had painful aching in mouth and 127 (83.6%) had difficulty during eating. Least impact was seen in alteration of taste 35 (23%).

**Conclusions:**

The pain intensity experienced by patients was variable. Most participants had moderate pain but few patients perceived no pain at all. The participants had at least one or other oral health impacts due to fixed orthodontic treatment. Orthodontists should counsel the patients regarding possible discomfort so that there is no discontinuation of treatment due to pain.

## INTRODUCTION

Oral health-related quality of life (OHRQoL) is defined as “a multidimensional construct that reflects (among other things) people’s comfort when eating, sleeping, and engaging in social interaction; their self-esteem; and their satisfaction with respect to their oral health.”^1^ Pain is one of the most important dimensions of oral health-related quality of life.^2^ Pain during orthodontic treatment is produced due to different.

Procedures such as separator placement, archwire placement and activations, application of orthopaedic forces, and debonding.^3^

Perception of pain is subjective showinglarge individual variations.^4^ Pain induced by orthodontic treatment is generally mild and short-lasting. However, some patients experience severe pain that may even impair mastication and tooth brushing.^5^

Therefore, this study was conducted to assess pain and oral health-related quality of life among patients undergoing orthodontic treatment in Kathmandu Medical College (KMC), Duwakot, Nepal.

## METHODS

A descriptive cross-sectional study was conducted in the Department of Orthodontics and Dentofacial Orthopaedics, KMC, Duwakot from January 2019 to October 2019 after receiving the ethical approval from Kathmandu Medical College and Teaching Hospital Institutional Review Committee (Ref. no. 2311201813). Informed consent was obtained from all the study participants before enrolling them in the study. A total of 152 patients undergoing fixed orthodontic treatment were chosen by convenience sampling method. Sample size was calculated using the formula:

n = Z^2^ × (p × q)/e^2^

    = 1.96^2^ × (0.9 × 0.1)/(0.05)^2^

    = 138.29

where,

n = required sample size
p = prevalence of pain due to orthodontic treatment (90%)^5^
q = 1-p
e = margin of error, 5%
Z = 1.96 at 95% Confidence Interval

Adding 10% for non-response= 138.29 + 13.82 = 152 approximately

Patients with ages ranging from 16 years to 30 years undergoing fixed orthodontic treatment with stainless steel brackets were included in the study. Patients who required any intraoral or extraoral appliances (e.g., bite plates, elastics), patients with severe Class II or Class III skeletal pattern who needed orthognathic surgery, syndromic patients, individuals with cleft lip or palate, and patients under medication for systemic diseases were excluded.

Pain during the first month of fixed orthodontic treatment was assessed using a numeric pain rating scale (NPRS). NPRS is a subjective measure in which individuals rate their pain on an eleven-point numerical scale. The scale ranged from 0 (no pain at all) to 10 (worst imaginable pain).^6^ Pain levels were rated as 0 = no pain; 1-3= mild pain (nagging, annoying, interfering little with activities of daily living); 4-6= moderate pain (interferes significantly with activities of daily living); 7-10= severe pain (disabling; unable to perform activities of daily living).

A face-to-face interview was done to record the oral health-related quality of life using Nepalese version of Oral Health Impact Profile (OHIP-14).^7^ OHIP-14 is a standard pretested questionnaire that has been translated and validated in different languages including the Nepali language. OHIP-14 consists of 14 items and explores seven dimensions of impact: functional limitation, physical pain, psychological discomfort, physical disability, psychological disability and handicap. The responses were coded as ‘very often’ (scoring 4), ‘fairly often’ (3), ‘occasionally’ (2), ‘hardly ever’ (1) or ‘never’ (0). The total score of OHIP-14 ranges from 0 to 56, which is calculated by summing the ordinal values for 14 items. The higher scores indicate increased oral health impact. The total OHIP-14 score and the subscale scores were used to measure the ‘severity’ of adverse impacts produced during the first month of fixed orthodontic treatment. In this study, each item of OHIP-14 scores was further dichotomised as “OHIP= 0 (i.e. no impact) and OHIP > 0 (i.e. impact on daily performance)” for cross-tabulation.

A pre-test was done among 25 patients with fixed orthodontic treatment before data collection for the main study. Inter-rater agreement for OHIP-14 was 0.99 indicating excellent reliability. Data obtained were entered in Microsoft Excel Sheet version 2007 and analysed using the Statistical Package for Social Sciences (SPSS version 11.5). The intra-class correlation coefficient (ICC) was used for determining interexaminer reliability in assessing OHIP-14. Descriptive statistics, including the mean and standard deviations, were computed for OHIP-14 and NPRS. Frequency distribution in the level of pain and OHIP-14 among the study population were calculated.

## RESULTS

Mean pain score of the study participants during the first month of fixed orthodontic treatment was 5.05±2.07 and their mean oral health impact was 12.71±7.27. Mean age of the study participants was 21.48±4.08 years. Among them, 55 (36.18%) were males and 97 (63.82%) were females. Out of 152 study participants, 37 (24.34%) experienced severe pain whereas 4 (2.63%) had no pain at all due to any procedures in fixed orthodontic treatment (Table 1). However, the study participants had experienced one or other adverse oral health impact while undergoing fixed orthodontic treatment (Table 2).

**Table 1:** Pain level during the first month of fixed orthodontic treatment.

Pain level	Male n (%)	Female n (%)	Overall participants’ response n (%)
0 (no pain at all)	3 (1.97)	1 (0.66)	4 (2.63)
1-3 (mild pain)	6 (3.95)	19 (12.5)	25 (16.45)
4-6 (moderate pain)	33 (21.71)	53 (34.87)	86 (56.58)
7-10 (severe Pain)	13 (8.55)	24 (15.79)	37 (24.34)

**Table 2:** Distribution of reported impacts in each item of OHIP-14.

Questions	Never n (%) Score 0	Hardly ever n (%) Score 1	Occasionally n (%) Score 2	Fairly Often n (%) Score 3	Very often n (%) Score 4	Overall impact (score 1-4)
Trouble pronouncing any words	79 (52)	25 (16.4)	40 (26.3)	7 (4.6)	1 (0.7)	73 (48)
Sense of taste has worsened	117 (77)	14 (9.2)	14 (9.2)	5 (3.3)	2 (1.3)	35 (23)
Painful aching in mouth	18 (11.8)	18 (11.8)	90 (59.2)	25 (16.4)	1 (0.7)	134 (88.2)
Uncomfortable to eat any food	25 (16.4)	22 (14.5)	68 (44.7)	31 (20.4)	6 (3.9)	127 (83.6)
Felt self-conscious	50 (32.9)	35 (23.0)	57 (37.5)	7 (4.6)	3 (2.0)	102 (67.1)
Felt tense	88 (57.9)	30 (19.7)	23 (15.1)	9 (5.9)	2 (1.3)	64 (42.1)
Diet been unsatisfactory	91 (59.9)	18 (11.8)	30 (19.7)	11 (7.2)	2 (1.3)	61 (40.1)
Had to interrupt meals	64 (42.1)	39 (25.7)	30 (19.7)	15 (9.9)	4 (2.6)	88 (57.9)
Difficult to relax	95 (62.5)	24 (15.8)	29 (19.1)	4 (2.6)	0 (0)	57 (37.5)
Been a bit embarrassed	95 (62.5)	25 (16.4)	23 (15.1)	5 (3.3)	4 (2.6)	57 (37.5)
Been a bit irritable with others	117 (77.0)	18 (11.8)	15 (9.9)	1 (0.7)	1 (0.7)	35 (23)
Had difficulty doing usual jobs	69 (45.4)	49 (32.2)	30 (19.7)	3 (2.0)	1 (0.7)	83 (54.6)
Life in general was less satisfying	100 (65.8)	27 (17.8)	21 (13.8)	2 (1.3)	2 (1.3)	52 (34.2)
Been totally unable to function	59 (38.8)	41 (27.0)	41 (27.0)	10 (6.6)	1 (0.7)	93 (61.2)

## DISCUSSION

The experience of pain and discomfort is a common situation during orthodontic treatment.^8^ Fixed orthodontic treatment provides the force to the tooth that produces prolonged pressure resulting in the acute inflammatory response. This leads to common sequelae of pain and discomfort to the patients.^9^ Studies have reported different impacts of orthodontic pain on oral health-related quality of life in patients undergoing fixed orthodontic treatment.^5,10^ Therefore, this study was conducted to assess pain and oral health-related quality of life among patients undergoing fixed orthodontic treatment in a dental college of Nepal.

The mean pain score in this study rated by NPRS was 5.05±2.07 indicating moderate pain perceived by the study participants during the first month of their fixed orthodontic treatment. Moderate pain experienced by the participants indicated that it interfered significantly with activities of their daily living.

Pain due to orthodontic treatment may be more severe than our expectations. Usually, treatment providers underestimate their patients’ pain and may not always know when analgesics are needed.^11^ In a study from India, 62% of dental practitioners and dental students predicted that the patients during the course of orthodontic treatment anticipate moderate pain and discomfort. However, moderate pain was experienced by 94% of orthodontic patients.^12^ In the current study, there were 86 (56.6%) participants who experienced moderate pain. Many (37, 24.3%) perceived severe pain as well, because of which they were unable to perform activities of daily life.

Most of the studies done to assess the impact of orthodontic treatment in oral health-related quality of life have reported that patients were considerably compromised in terms of their overall OHRQoL until approximately one month after appliance insertion.^13^ In this study, the overall impact of fixed orthodontic treatment during the first month of treatment was 12.71±7.27. The individuals had variable impacts on their oral health-related quality of life due to the commencement of fixed orthodontic treatment. This finding is similar to a study done in Brazil where patients experienced worsened quality of life during orthodontic treatment because of functional limitations and physical pain.^14^ Not a single participant in the current study was found to be devoid of any impact. This finding was similar to the study done in Switzerland where almost every patient reported some amount of interference in schoolwork or social activities.^15^

Most of the study participants had painful aching in the mouth (134, 88.2%) and difficulty in having food (127, 83.6%). Similar frequency of pain and discomfort due to fixed orthodontic treatment were observed in a study done in India and Pakistan.^5,10^ Likewise, in a study done in Lithuania, patients wearing braces complained of impaired nutrition more than patients wearing removable and functional appliances.^18^ However, a study done in Michigan showed that only 21.9% of the patients changed their diet due to pain from the braces.^11^

In a study done in Lithuania, it was observed that 26.8% of the patients had discomfort due to impaired speech. However, in this study, more 73 (48%) participants had difficulty in pronouncing words.^16^ Total of 83 (54.6%) patients in this study reported to have difficulty in doing usual jobs, 57 (37.5%) felt a bit embarrassed and 93 (61.2%) were completely unable to function due to pain and discomfort from fixed orthodontic treatment. This finding is consistent with the study done in China, where physical functioning, body pain and general mental health domains were affected after initial archwire placement indicating patients’ lives being affected by orthodontic treatment.^17^ These results help orthodontists to inform patients about the likely impact of fixed orthodontic treatment so that the possible pain and discomfort may be accepted by them once they start the treatment.

Pain and its influence on daily life can be the major cause for discontinuation of orthodontic treatment.^15^ Social discomfort due to fixed orthodontic treatment is more during the initial stages of treatment. Physiological adaptation occurs with time, the speed of which is dependent upon personality characteristics of the patient. The orthodontist should make primary consideration to this matter as an early intervention might help prevent the long-lasting establishment of a stereotype denial of orthodontic appliance.^18^

The study has some limitations. This study is done in a small sample at a single dental hospital which cannot be generalised to all the fixed orthodontic treatments done in Nepal. The study should be conducted in a larger population to elucidate the effect of orthodontic pain on quality of life. The study records pain and quality of life only in the first month of commencement of fixed orthodontic treatment and using only a single wire type. Longitudinal analytical studies assessing pain and quality of life in different orthodontic treatment phases, as well as different wire types, can better evaluate the changes in pain perception and quality of life following orthodontic treatment.

## CONCLUSIONS

The pain intensity experienced by patients in the first month of fixed orthodontic treatment in Kathmandu Medical College, Duwakot, Nepal was variable. More than half of the patients had moderate pain and few patients perceived no pain at all. The study participants had at least one or other oral health impact due to fixed orthodontic treatment. Hence, it is recommended that orthodontists should give more time to communicate and counsel the patients regarding possible discomfort so there are no discontinuations of treatment due to pain.
